# Poisoning among Patients Presenting to the Department of Emergency Medicine of a Tertiary Care Centre: A Descriptive Cross-sectional Study

**DOI:** 10.31729/jnma.7878

**Published:** 2022-10-31

**Authors:** Kabir Thakali, Nistha Ulak, Manjil Bharati, Lok Jung Thapa, Dipendra Nepali Paudyal, Chandra Kiran Basnet, Dinesh Kumar Lamsal, Ramesh Pant

**Affiliations:** 1 Department of General Practitioners and Emergency Medicine, Nepalese Army Institute of Health Sciences, Sanobharyang, Kathmandu, Nepal; 2 Ramghat Primary Health Center, Gaushala, Kathmandu, Nepal; 3 Himal Hospital, Gyaneshwor, Kathmandu, Nepal; 4 Meditech Hospital, Siddartha Rajmarg, Butwal, Nepal, Gyaneshwor, Kathmandu, Nepal; 5 P.T. Birta City Hospital And Research Centre, Birtamod, Jnapa, Nepal; 6 Department of Family and Emergency Medicine, Civil Service Hospital of Nepal, New Baneshwor, Kathmandu, Nepal

**Keywords:** *emergency department*, *poisoning*, *prevalence*

## Abstract

**Introduction:**

Poisoning is one of the major public health problems worldwide. The World Health Organization has estimated 0.3 million deaths a year due to various poisoning agents; pesticides being the leading one. Poisoning is one of the leading causes of emergency room visits. It has become a significant public health issue in Nepal. This study aimed to find out the prevalence of poisoning among patients presenting to the Department of Emergency Medicine of a tertiary care centre.

**Methods:**

A descriptive cross-sectional study was done in the Department of Emergency Medicine of a tertiary care centre from 24 June 2022 to 6 July 2022. Ethical approval was obtained from the Institutional Review Committee (Reference number: 11/2022). Data from 765 patients were collected from the hospital records. The patient's history and clinical examination were used to make the diagnosis of poisoning. Convenience sampling method was used. Point estimate and 95% Confidence Interval were calculated.

**Results:**

Among 765 patients visiting the emergency department, 8 (1.04%) (0.99-1.09, 95% Confidence Interval) patients were of poisoning. The most common poison used was organophosphates which was consumed by 2 (25%) patients and benzodiazepines by 2 (25%) patients.

**Conclusions:**

The prevalence of poisoning among patients presenting to the Department of Emergency Medicine was similar when compared to other studies conducted in similar settings. Although agricultural poisons were commonly used, cases of drug poisoning seems to be on the rise nowadays.

## INTRODUCTION

Poisoning is one of major public health problem worldwide and World Health Organization (WHO) estimated 0.3 million people die every year due to various poisoning agents.^1^ Worldwide various agents such as agrochemicals, drugs or environmental agents are used as poisoning agents.^2^ Intentional poisoning is one of the important causes for mortality and morbidity worldwide.^3^

Poisoning is one of the major causes of hospitalization through emergency and is a major public health problem in Nepal.^4^ All the cases of poisoning are admitted through emergency services where the safety of life of the patient is the main issue for doctor.^5^

This study aimed to find out the prevalence of poisoning among patients presenting to the Department of Emergency Medicine of a tertiary care centre.

## METHODS

A descriptive cross-sectional study was done in the Department of Emergency Medicine of Civil Service Hospital from 24 June 2022 to 6 July 2022. Ethical approval was obtained from the Institutional Review Committee (Reference number: 11/2022). All the patients presenting to the department within the study period were included in the study. Missing data and incomplete hospital records were excluded. Convenience sampling method was used. The sample size was calculated using the following formula:


$$\begin{array}{ll} \text{n} & ={\text{Z}}^{2}\times \frac{\text{p}\times \text{q}}{{\text{e}}^{\text{2}}}\\ & =1.96^{2}\times \frac{0.50\times 0.50}{0.05^{2}}\\ & =385 \end{array}$$

Where,

n= minimum required sample size
Z= 1.96 at 95% Confidence Interval (CI)
p= prevalence taken as 50% for maximum sample size calculation
q= 1-p
e= margin of error, 5%

A sample size of 385 was calculated. Ten percent was added to address the missing data after which the minimum required sample size was 427. However, a sample size of 765 was taken. After obtaining permission from the emergency department of the same hospital, the essential information was gathered from the hospital records. The diagnosis of the poisoning was made according to the patient's history, clinical examination and laboratory findings.

Data on the diagnosis, age, gender, trends of poisoning, chronological findings and outcome were collected. Data were entered and analysed by Microsoft Excel 2016. Point estimate and 95% CI were calculated.

## RESULTS

Among 765 patients visiting the emergency department, eight (1.04%) (0.99-1.09, 95% CI) patients were of poisoning (Table 1).

**Table 1:** Socio-demographic findings (n= 8).

Parameters	Number of cases n (%)
**Age group (years)**	
11-20	5 (62.50)
21-30	1 (12.50)
31-40	1 (12.50)
>40	1 (12.50)
**Gender**	
Males	5 (62.50)
Females	3 (37.50)

The most common poison used was organophosphates which was consumed by 2 (25%) patients and benzodiazepines by 2 (25%) patients. A total of 6 (75%) of the poisoining cases were intentional and oral route of poison intake was seen in 8 (100%) of the patients (Table 2).

**Table 2:** Details of the poisoning (n= 8).

Parameters	Number of cases n (%)
**Manner of poisoning**	
Intentional	6 (75)
Accidental	2 (25)
Homicidal	-
**Type of poison**	
Organophosphorus	2 (25)
Benzodiazepine	2 (25)
Aluminum phosphate	1 (12.50)
Multidrug overdose	1 (12.50)
Honey	1 (12.50)
Cypermethrin	1 (12.50)

A total of 4 (50%) of the cases presented to the hospital within 4 hours of poison intake, 3 (37.5%) were unconscious, 4 (50%) stayed at the hospital for 4-6 hours and 4 (50%) of the cases left against medical advice (Table 3).

**Table 3:** Chronological details (n= 8).

Parameters	Number of cases n (%)
**Time of hospital arrival after poison intake (hours)**	
Within 4	4 (50)
After 4	4 (50)
**Condition of the patient at the time of presentation**	
Unconscious	3 (37.50)
Conscious	5 (62.50)
**Hours of stay (hours)**	
0-2	2 (25)
2-4	2 (25)
4-6	4 (50)
**Outcome**	
Shifted to the Intensive Care Unit	3 (37.50)
Referred to other centres	1 (12.50)
Left against medical advice	4 (50)

A total of 7 (87.50%) patients received intravenous fluids, 4 (50%) of the cases required nasogastric tube insertion and gastric lavage, 5 (62.50%) were treated with proton pump inhibitors, 3 (37.50%) received atropine and 4 (50%) received ondansetron during the treatment at the Department of Emergency Medicine.

## DISCUSSION

Poisoning remains a public health problem with significant morbidity and mortality and is one of the major causes for emergency visit and hospital admission.^6^ Prompt diagnosis and appropriate management is crucial for better clinical outcome.

In this study, majority of the cases of poisoning were of age group 11-20 years (62.5%), which was similar to that found in a study done in Nepal where the most common age group was 15-25 years (50.1%).^7^ Psycho-emotional problems common to this age group include academic failure, unemployment, financial difficulties, failed romantic relationships, domestic pressure, and others. Such problems cause people to have a pessimistic outlook on life and are positively correlated with suicidal attempts.^8^

Our study showed male predominance in poisonings with female to male ratio 0.6:1 which was different to the other studies done in Nepal where female predominance was found.^9,10^ Female to male ratio was found to be 1.3:1 in study done in five major hospitals of Nepal and 1.6:1 in Nepal Medical College Teaching Hospital, Kathmandu.^10,11^ Since all patients with poisoning that were admitted to the Emergency at Civil Hospital were only included in the study population, the actual gender ratio might differ when all the patients that are admitted to emergency care are included.

Consistent with other studies, majority of cases (62.5%) were conscious and 37.5% cases were unconscious at the time of arrival to hospital which is comparable to teaching hospital in western Nepal where 59.18% cases were conscious, 22.45% semi-conscious and 13.27% unconscious at the time of hospital admission.^12^ The general condition of the patients plays an important role in managing the patients. In general, the use of CNS depressants and induction of vomiting is better avoided in unconscious patients and in patients with poor Glasgow Coma Scale (GCS) score are to be intubated to prevent aspiration.

Half of the cases (50%) arrived hospital within four hours after exposure to poison which is similar to study done in teaching hospital in western Nepal where 48.98% cases arrived hospital within four hours of poison exposure and study done in Bir Hospital where 61.9% cases presented within three hours of poison exposure.^12,13^

Majority of cases of poisoning were found to be intentional poisoning in our study (75%) followed by accidental poisoning (25%) which is different from the study done in Bir Hospital reveals that 97 (98.0%) cases were intentional poisoning for suicidal attempt and 2 (2.0%) cases had accidental poisoning.^13^ But the study done in Dhulikhel hospital had similar findings where 77.78% of the patients consumed poison intentionally and rest of the cases 22.23% consumed accidentally.^14^ And, as expected, the route of exposure and place of exposure in most cases were oral and home respectively.

The present study revealed that amongst the type of poison, organophosphorus poisoning and Benzodiazepine both constitute the commonest cause 25%, followed by Aluminium phosphate , multidrug poisoning , honey and cypermethrin 12.5% each. This findings shows that people are using drugs and other forms of poison rather than majority of people using organophosphate poison which is in contrast to the findings done in other studies where organophosphate poisoning constituted the majority of cases.^13-5^ Upon occurrence of drug poisoning several parameters should be considered by the clinicians while managing the patients. It includes the protein binding, plasma half-life, peak plasma concentration etc. In Nepal, even narcotics and psychotropic drugs can be purchased from a pharmacy without having a prescription.

Our study showed that 37.5% cases were shifted to ICU, 12.5% were referred to other center and 50% left against medical advice which is different from the study done in Kathmandu medical college where about 37.3% of the poisoning cases were discharged from Emergency after emergency management and observation for few hours, 50.7% cases were admitted to ICU/medical ward and one patient (1.5%) died in emergency while undergoing treatment.^15^ As our study was done in Emergency department only, half of cases were found to be discharged or referred to ICU within four hours of presentation on Emergency ward whereas other half of the cases stayed between 4 to 6 hours.

The common drugs used in the management of poisoning were related to gastrointestinal and parental fluids were administered in most of the cases. The gastrointestinal agents used were mainly proton pump inhibitors to prevent acid secretion. We encountered use of antiemetic in 25% of the cases during the course of treatment. Gastric lavage is indicated for patients who ingested potentially lethal amount of toxins. It has the greatest benefit if administered within 1 hour of ingestion.^16^ In our study, half of the cases underwent gastric lavage. Atropine was used in all the patients with OP poisoning. As most of the cases of poisoning are suicidal in nature, all patients with poisoning must be given psychiatric assessment during their stay in the hospital and also at the time of discharge. If possible, follow up visits should also be planned.

Because this study was limited to a single hospital, its findings cannot be applied to the entire nation. Therefore, a population-based investigation is required to ascertain the exact degree of poisoning exposure and intoxication.

## CONCLUSIONS

The prevalence of poisoning among patients presenting to the Department of Emergency Medicine was similar when compared to other studies conducted in similar settings. Drug poisoning seems to be on the rise nowadays. Psychiatry consultation is required for patients who have intentionally poisoned themselves while they are hospitalized in order to reduce the likelihood that they would attempt self harm again. Despite medical advise, nearly half of patients tend to leave the hospital as soon as their medical condition begins to modestly improve without completing their course of treatment and required supervision. Strict rules must be followed regarding sale of pesticides and sedatives.
